# Ultrastructural and diffusion tensor imaging studies reveal axon abnormalities in Pompe disease mice

**DOI:** 10.1038/s41598-020-77193-w

**Published:** 2020-11-19

**Authors:** Ni-Chung Lee, Wei-Hao Peng, Li-Kai Tsai, Yen-Hsu Lu, Hao-Chun Wang, Yao-Chia Shih, Zeng-Xian Pung, Hsi-Yuan Hu, Wuh-Liang Hwu, Wen-Yih Isaac Tseng, Yin-Hsiu Chien

**Affiliations:** 1 Department of Medical Genetics, National Taiwan University Hospital, Taipei, 10041 Taiwan; 2 Department of Pediatrics, National Taiwan University Hospital and National Taiwan University College of Medicine, Taipei, 10041 Taiwan; 3 School of Medicine for International Students, College of Medicine, I-Shou University, Kaohsiung, 84001 Taiwan; 4 Department of Neurology, National Taiwan University Hospital, Taipei, 10041 Taiwan; 5 Department of Medical Imaging, National Taiwan University Hospital, Taipei, 10041 Taiwan; 6 Institute of Medical Device and Imaging, National Taiwan University College of Medicine, Taipei, 10041 Taiwan; 7 Duke-Nus Medical School, Singapore, 169857 Singapore

**Keywords:** Medical genetics, Neurology

## Abstract

Pompe disease (PD) is caused by lysosomal glycogen accumulation in tissues, including muscles and the central nervous system (CNS). The intravenous infusion of recombinant human acid alpha-glucosidase (rhGAA) rescues the muscle pathologies in PD but does not treat the CNS because rhGAA does not cross the blood–brain barrier (BBB). To understand the CNS pathologies in PD, control and PD mice were followed and analyzed at 9 and 18 months with brain structural and ultrastructural studies. T2-weighted brain magnetic resonance imaging studies revealed the progressive dilatation of the lateral ventricles and thinning of the corpus callosum in PD mice. Electron microscopy (EM) studies at the genu of the corpus callosum revealed glycogen accumulation, an increase in nerve fiber size variation, a decrease in the g-ratio (axon diameter/total fiber diameter), and myelin sheath decompaction. The morphology of oligodendrocytes was normal. Diffusion tensor imaging (DTI) studies at the corpus callosum revealed an increase in axial diffusivity (AD) and mean diffusivity (MD) more significantly in 9-month-old PD mice. The current study suggests that axon degeneration and axon loss occur in aged PD mice and are probably caused by glycogen accumulation in neurons. A drug crossing the BBB or a treatment for directly targeting the brain might be necessary in PD.

## Introduction

Pompe disease (PD; MIM #232300) is an inherited disorder in which a deficiency in acid α-glucosidase (GAA; EC 3.2.1.20) causes the intralysosomal accumulation of glycogen, most notably in skeletal and cardiac muscles^1^. PD patients present with a wide spectrum of phenotypes, ranging from the rapidly fatal infantile-onset PD (IOPD) to other later-onset forms. Enzyme replacement therapy (ERT) with recombinant human GAA (rhGAA)^2,3^ effectively reverses cardiomegaly in patients with IOPD^4,5^, and initiating ERT immediately after the detection of IOPD by newborn screening further improves the outcome of skeletal muscles in young children with IOPD^6,7^. Although slow-progressing myopathy, including ptosis and speech disorders, still develops starting from the ages of 3–5 years in the early-treated IOPD patients, the survival of patients is significantly prolonged^8–12^. The prolonged survival of IOPD patients raises a concern regarding central nervous system (CNS) involvement in IOPD^11–13^ because rhGAA does not penetrate the blood–brain barrier.

Glycogen accumulation in the brain stem and spinal cord motor neurons, Schwann cells, and oligodendrocytes was observed in autopsies of PD patients^14–16^. Cranial nerve dysfunction in IOPD patients may include swallowing abnormalities, sensorineural hearing loss^9^, speech articulation disorders^9^, lingual weakness^17^, and respiratory insufficiency^14^. In a *Gaa* knock-out mouse model, extensive glycogen accumulation was observed in both neurons and glial cells in the cortex, cerebellum, brain stem, and spinal cord^18^. The swollen motor neurons contained numerous glycogen-filled vacuoles^19^. Transcriptome analysis of the spinal cord revealed that both cell death and proinflammatory signaling pathways were upregulated^20^. Astrogliosis has also been observed in old PD mice^18^. We demonstrated that neuron-specific gene therapy can relieve motor deficits in PD mice^21^.

In the early period of launching ERT, we also described delays in myelination milestones in five untreated patients with IOPD at a median age of 6 months, although after ERT, four of the five patients showed improvements in myelination^22^. However, after several years (ERT starting at a median age of 16 days for a median time of 63 months), we found hypomyelination in early-treated IOPD patients that manifested as progressive T2-weighted hyperintense signals on brain magnetic resonance imaging (MRI)^12^. In another study on IOPD, brain MRI abnormalities were found over the periventricular white matter, corpus callosum, internal capsule, brainstem, etc.^13^. We also observed prominent astrogliosis and hypomyelination in PD mice, and the changes were improved by neuronal-specific gene therapy^21^. Unfortunately, we still do not understand the pathogenesis of white matter abnormalities in PD.

Although studies have described CNS pathologies in PD, the reported patients were heterogeneous in disease severity, age at starting treatment, age at evaluation, and complications, including chronic ventilator use, hypoxia, or vascular lesions. PD mouse models have been informative regarding the role of CNS pathologies in PD, but a detailed study of white matter is still lacking. Therefore, we designed an experiment to study age-related white matter changes in PD mice.

## Results

### Progressive thinning of the corpus callosum in PD mice

**Figure 1:**
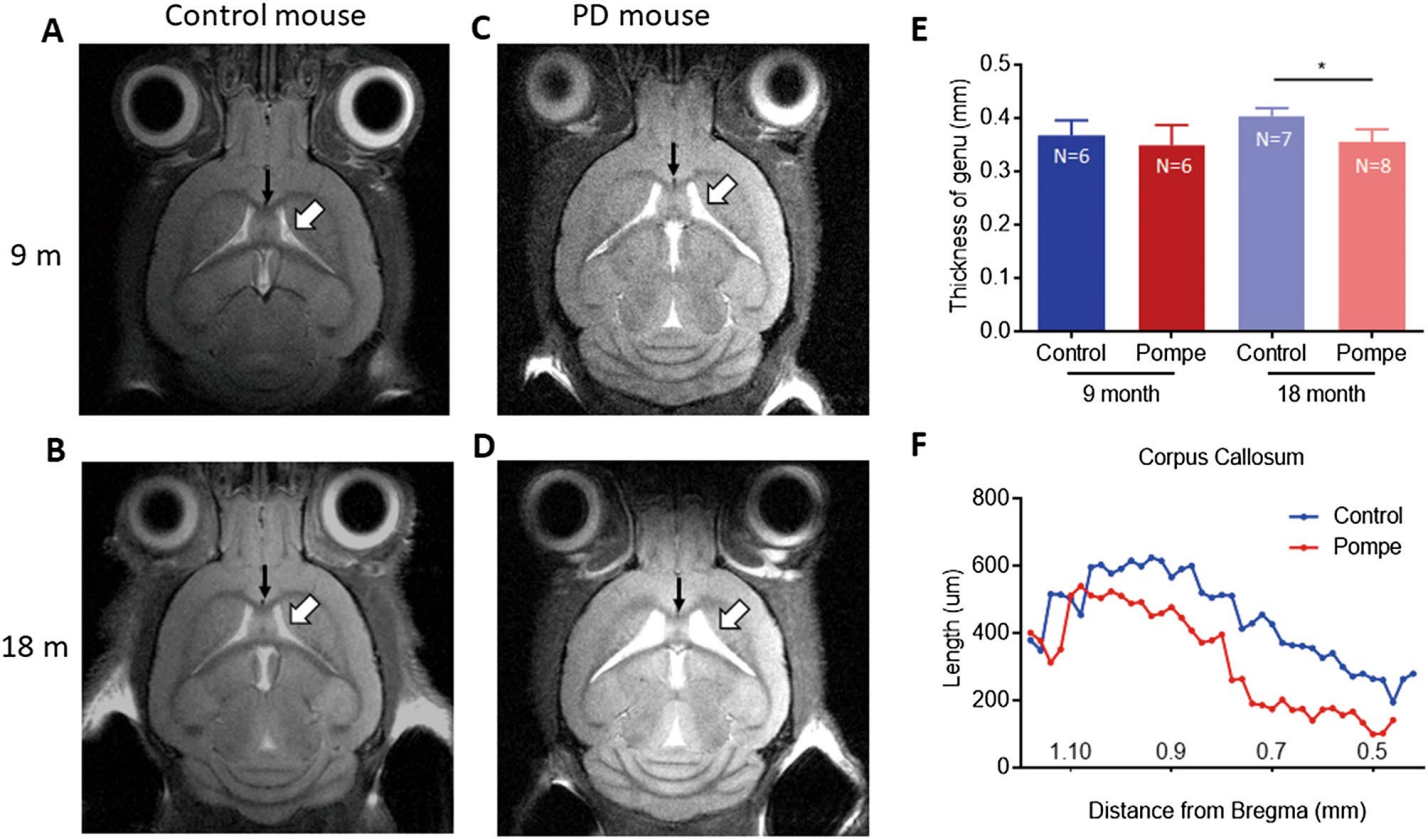
Structural changes in PD mice. (**A**–**D**) Axial T2-weighted MRI images obtained from 9- to 18-month-old mice revealed the progressive dilatation of the lateral ventricles (white arrows) and thinning of the corpus callosum (black arrows) in PD mice. The relative T2 signal intensity ratio over the corpus callosum was not different in PD mice (*p* = 0.631 and 0.165 in the 9- and 18-month groups, respectively, by the Mann–Whitney test). (**E**) The thickness at the genu of the corpus callosum was calculated and compared between PD and control mice. *Indicates *p* < 0.001. (**F**) The thickness of the corpus callosum in a pair of control and PD mice was measured along the craniocaudal axis through serial coronal sections and H&E staining.

The 9-month-old PD mice had no difference in body weight from the control mice. The 18-month-old PD mice had a decreased body weight than the 18-month-old control mice (*p* < 0.001, Mann–Whitney test) or the 9-month-old PD mice (*p* = 0.004, Mann–Whitney test). T2-weighted axial MRI images were first obtained in 9-month-old male control and PD mice (control: n = 6; PD: n = 6). PD mice exhibited mild dilatation of lateral ventricles (Fig. 1A,C, white arrows), but the thickness of the corpus callosum was not abnormal (Fig. 1E). Four control mice and two PD mice that had been tested at 9 months of age were alive at 18 months of age, and 3 more control mice and 6 more PD mice were added to the 18-month analysis (total control: n = 7; PD: n = 8). Control mice did not show any change over the time interval (Fig. 1A,B), whereas progressive dilatation of the lateral ventricles was observed in PD mice (Fig. 1C,D, white arrows). The genu of the corpus callosum was also thinner in PD mice than in control mice (*p* = 0.0004, unpaired t test; Fig. 1E). The T2 signal intensity ratio over the corpus callosum was not different between the control and PD mice (*p* = 0.631 and 0.165 for the 9- and 18-month groups, respectively, Mann–Whitney test), suggesting that there were no gross hypomyelination on T2-weighted images in PD mice. The brains of a pair of mice were fixed and subjected to serial coronal sections and hematoxylin and eosin (H&E) staining. The results showed that the corpus callosum was thin throughout its longitudinal span in the PD mouse (Fig. 1F).

### Ultrastructural abnormalities of the corpus callosum in PD mice

**Figure 2:**
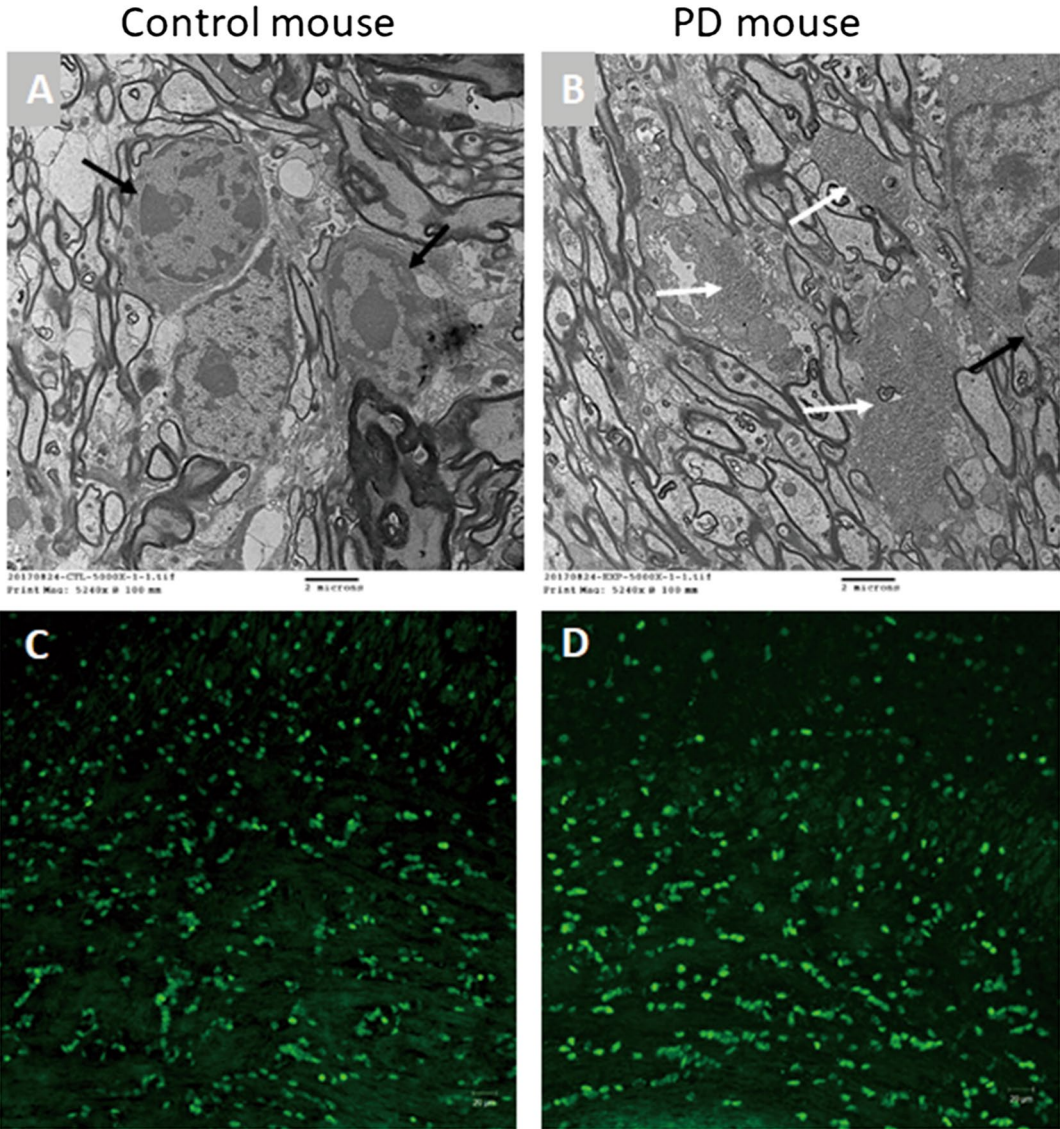
Ultrastructure of the corpus callosum in 9-month-old mice. (**A**) A representative image from the control mouse shows two oligodendrocytes (black arrows), and there was no glycogen accumulation in the image. (**B**) A representative image from the PD mouse shows glycogen accumulation (white arrows) between the myelinated axons but normal cytoplasm in the oligodendrocyte (black arrow) on the right. (**C**) Immunohistochemical staining with an anti-Oligo2 antibody (green) shows the distribution of oligodendrocytes in the corpus callosum in a control mouse. (**D**) Oligo2 staining in a PD mouse shows the same pattern as in the control mouse.

**Figure 3:**
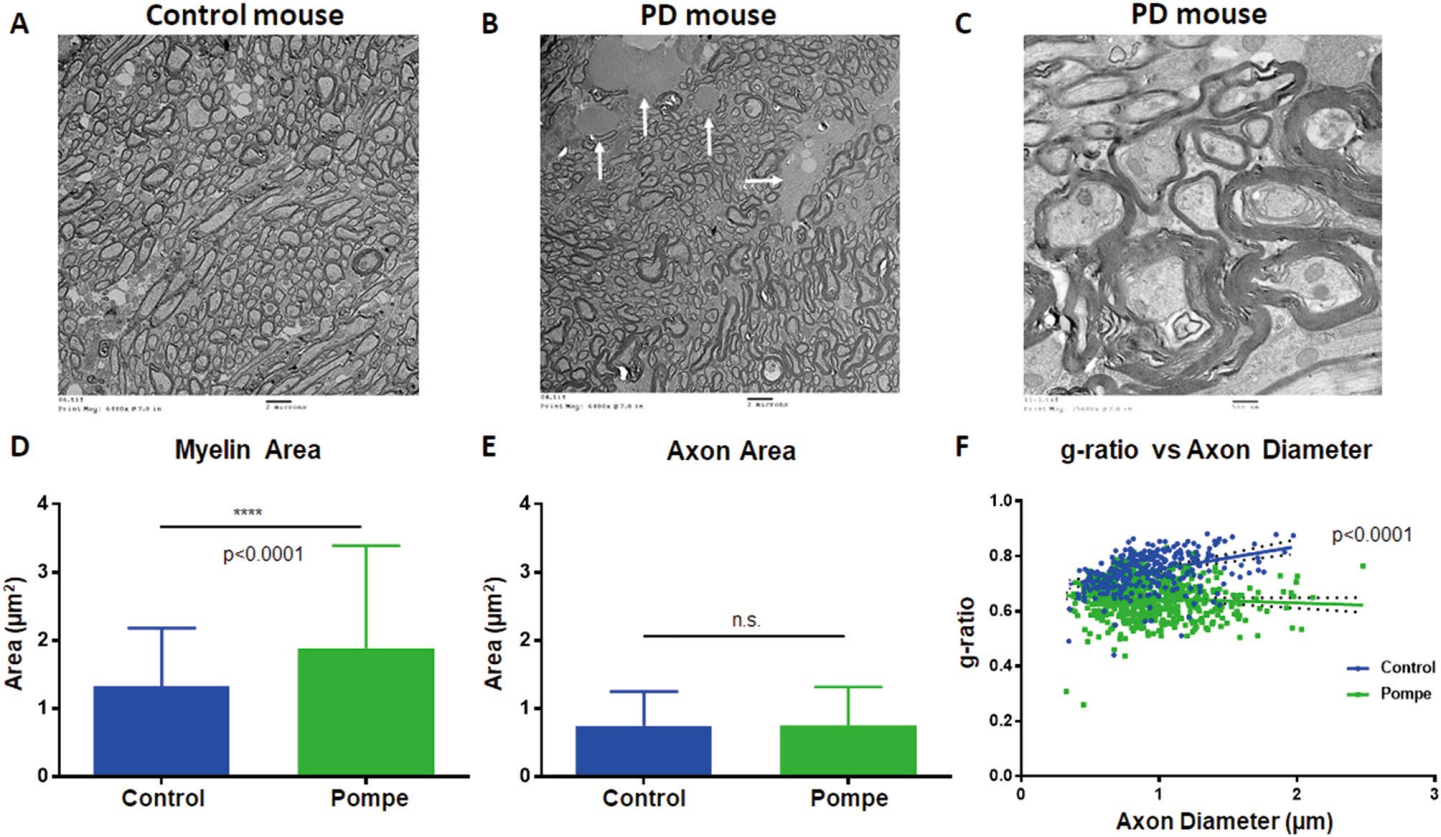
Ultrastructure at the genu of the corpus callosum in 18-month-old mice. (**A**) A representative low-magnification image from control mice. (**B**) A representative low-magnification image from a PD mouse shows glycogen accumulation, an increase in nerve fiber size variation, and groups of large fibers with a thickened myelin sheath. (**C**) A high magnification image from a PD mouse shows large nerve fibers with a thickened myelin sheath, splitting of the myelin sheath, and disruption of mitochondrial cristae. (**D**) The myelin area was larger in PD mice (*p* < 0.0001). (**E**) There was no difference in the mean axon area between control and PD mice (*p* = 0.676). (**F**) The axon g-ratio/diameter plot revealed that the g-ratio did not increase with the diameter in PD mice.

The genu of the corpus callosum, lateral portion, of 18-month-old control and PD mice was examined by electron microscopy (EM) (Fig. 2A,B). In the PD mice, glycogen-filled lysosomes and vacuoles were frequently found in the cell processes (Fig. 2B, white arrows) and cell bodies of astrocytes but were not found in the cell bodies of oligodendrocytes (Fig. 2B, black arrow). We also employed immunohistochemical staining to examine the distribution of oligodendrocytes in the corpus callosum, and the results exhibited a similar pattern for control and PD mice (Fig. 2C,D). Morphologically, PD mice had a greater variation in nerve fiber sizes (Fig. 3A,B). Groups of large, irregular, nerve fibers with thickened myelin sheaths were frequently observed in the PD mice (Fig. 3B, right lower corner), and high magnification images revealed the splitting of the myelin sheath and disruption of mitochondrial cristae (Fig. 3C). There was no splitting of myelin sheath in the control mice (Fig. S2). The myelin area of PD mice (1.891 ± 1.502) was significantly larger than control mice (1.328 ± 0.854; *p* < 0.0001; Fig. 3D), and the coefficient of variation was also larger in PD mice (0.79) than in control mice (0.64). The axon area did not differ between control (0.749 ± 0.502) and PD mice (0.756 ± 0.561; *p* = 0.676; Fig. 3E). The g-ratio (axon diameter/myelin diameter) was smaller in PD mice than in control mice (*p* < 0.0001; Fig. 3F).

### Diffusion tensor imaging changes in PD mice

Diffusion tensor imaging (DTI) was performed on control and PD mice, and the analyzed parameters included fractional anisotropy (FA), axial diffusivity (AD, longitudinal diffusivity), radial diffusivity (RD), and mean diffusivity (MD). One each region of interest (ROI) was selected for the medial, left lateral, and right lateral corpus callosum, and data from the lateral corpus callosum were averaged. The 9-month-old PD mice exhibited higher AD, RD, and MD values over the lateral corpus callosum, and higher AD and MD values over the medial corpus callosum, than control mice (Table 1). The 18-month-old PD mice only exhibited a higher AD value over lateral corpus callosum than control mice. There was no difference in FA between the Pompe and control mice.

**Table 1:** The AD, RD, MD, and FA values over the lateral and medial corpus callosum (CC) in the 9- and 18-month-old control and Pompe disease mice.

	9 m	Control mice	Pompe mice	*p* value	18 m	Control mice	Pompe mice	*p* value
Lateral CC	AD	0.827 (0.042)	0.874 (0.052)	0.023*	AD	0.781 (0.037)	0.838 (0.070)	0.023*
	RD	0.650 (0.024)	0.688 (0.064)	0.014*	RD	0.606 (0.041)	0.640 (0.067)	0.219
	MD	0.710 (0.027)	0.751 (0.058)	0.010*	MD	0.667 (0.041)	0.706 (0.067)	0.204
	FA	0.280 (0.034)	0.278 (0.036)	0.818	FA	0.302 (0.023)	0.309 (0.023)	0.395
Medial CC	AD	1.128 (0.082)	1.249 (0.032)	0.008*	AD	0.872 (0.072)	0.865 (0.046)	0.865
	RD	0.863 (0.035)	0.904 (0.065)	0.230	RD	0.597 (0.055)	0.609 (0.065)	0.865
	MD	0.961 (0.049)	1.027 (0.051)	0.031*	MD	0.690 (0.058)	0.693 (0.059)	0.952
	FA	0.351 (0.022)	0.343 (0.051)	0.575	FA	0.392 (0.022)	0.377 (0.024)	0.271

Values are expressed as mean (SD).

*p < 0.05.

## Discussion

We investigated the pathogenesis of white matter disease in PD by ultrastructural and diffusion tensor imaging studies in 9- and 18-month-old PD mice, at the time these mice were still reasonably healthy. This is in contrast to human brain studies in PD patients who were in the final stages of the disease and died of complications including asphyxia and chronic hypoxemia. Therefore, we can conclude that axon degeneration and axon loss occur in aged PD mice and are probably caused by glycogen accumulation in neurons.

Based on the T2-weighted hyperintense signals of the deep white matter in early-treated IOPD patients with long-term survival, a concern has been raised about CNS diseases^12^. However, T2-weighted hyperintense signals of the deep brain white matter that were previously noted in PD patients could be due to either glycogen- or demyelination-associated water retention (water exhibits high T2 intensity)^13^. We have followed a cohort of early-treated patients with glutaric aciduria type I, a disease of defective l-lysine metabolism that the accumulation of glutaric acid and other compounds damage the brain. In those patients, T2-weighted hyperintensity occurred in more than half of the patients when they reached school age, but their neurological status remained normal^23^. In the current study, we confirmed that there was glycogen accumulation in the corpus callosum, probably in the processes of astrocytes. Functionally, astrocytes in white matter promote and maintain myelination^24^, but there was no gross demyelination or dysmyelination in the PD mice.

Signs of axon degeneration found in the current study included the splitting of the myelin sheath and a decrease in the g-ratio. The axon of an unhealthy neuron can progressively degenerate, a process called “dying back”, beginning distally and slowly spreading toward the cell body^25^. It has been demonstrated in an animal model of amyotrophic lateral sclerosis, a motor neuron disease, that axonal pathology presents as a decompaction of myelin sheaths with a decreased g-ratio, an increase in large-diameter axons, and a decrease in axonal numbers in the ventrolateral white matter^26^. In PD mice, enlarged, swollen, degenerating axon terminals were observed in the lower brainstem and spinal cord where motor neurons are filled with glycogen vacuoles^18,19^, and hypermyelinated fibers, increase in nerve fiber size, and decrease in g-ratio also occurred in the phrenic nerve^27^.

**Figure 4:**
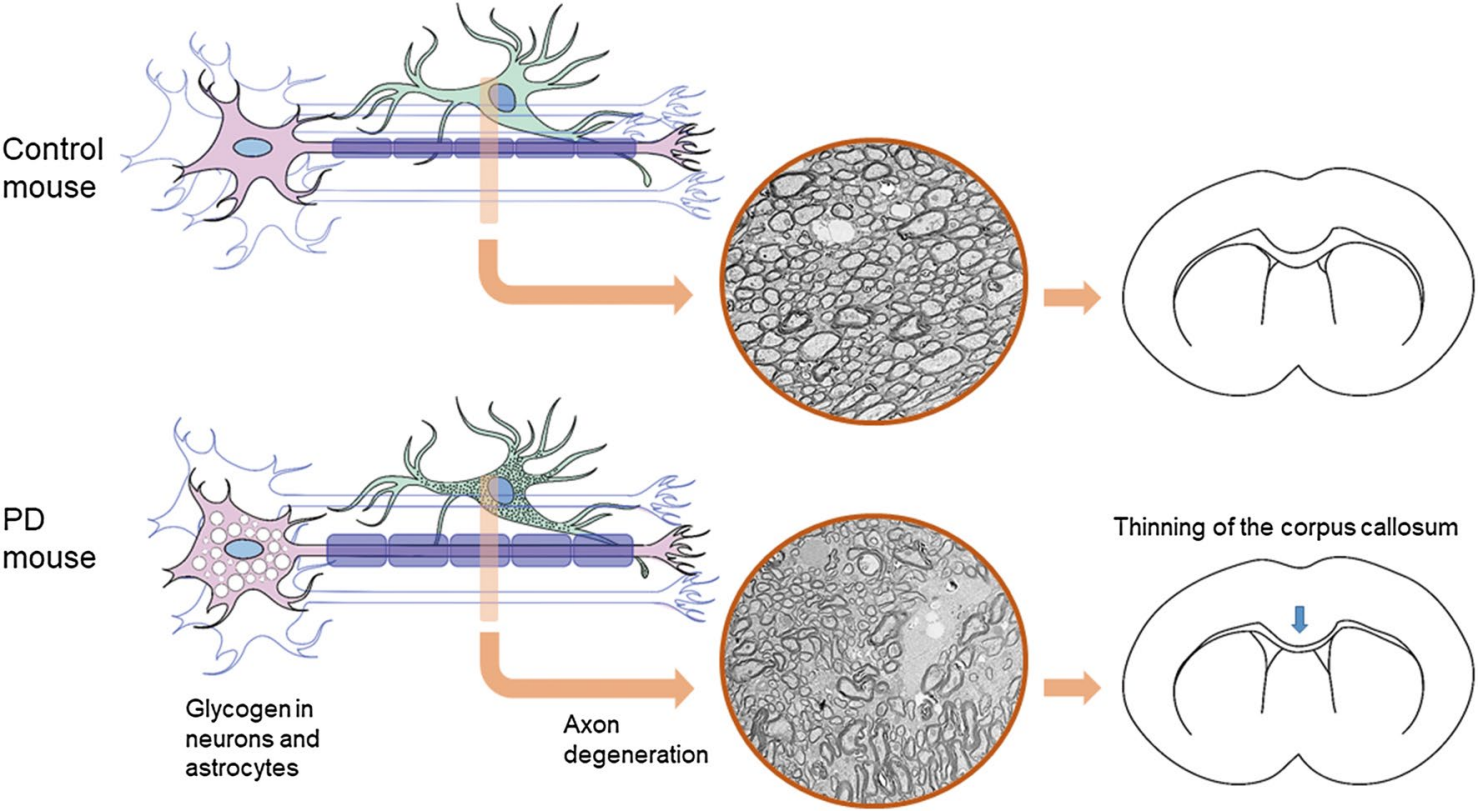
Proposed processes of white matter changes in PD. Glycogen accumulation in neurons (pink) and astrocytes (green) causes axon degeneration, and the process finally leads to the loss of white matter volume.

The interpretation of the DTI data^28^ was complicated by the combinatory effects of axon degeneration and glycogen accumulation (Fig. 4). AD and MD increased in the 9-month-old PD mice over the corpus callosum. AD is generally considered to indicate movement along the axon, and an increase in AD may be a result of the swelling of the axon in the PD mice. Therefore, changes in the axon should have occurred at 9 months of age in the PD mice. However, these DTI changes became less consistent in the old (18-month-old) PD mice that diseases had progressed. It is likely that when disease advanced in the old mice, glycogen increasingly accumulated in the corpus callosum. Glycogen binds to large quantity of water and may hinder the diffusion of water.

We carefully examined the corpus callosum in PD mice by EM and staining. Most of the glycogen accumulation in the corpus callosum occurred in astrocytes and their processes, and glycogen accumulated was never prominent in oligodendrocytes. Immunohistochemical studies also revealed a normal density of oligodendrocytes in the corpus callosum. Because there were no signs of hypomyelination on T2-weighted images, oligodendrocytes may not play a major role in the CNS pathology in PD. In conclusion, the progressive decrease in white matter volume with age in PD mice indicates that a process of axon degeneration occurs, which could be due to the impaired health of neurons in the cortex or nuclei that send out the axons.

A limitation of the current study was that we were unable to generate direct links between neuronal and axonal damage. Neuronal glycogen accumulation in PD has been well demonstrated and was not part of the current study. Tracing the damaged axons back to the corresponding neurons is, however, beyond the scope and capacity of the current study. Transgenic animals with neuron group-specific fluorescence labeling may be able to create a physical link to validate our hypothesis.

## Methods

### PD mice

Male PD mice were established by disrupting exon 6 in the *GAA* gene using a *neo* cassette^29^. These mice exhibited weakness beginning at 3.5 weeks. By 8–9 months of age, the animals developed obvious muscle wasting and a weak, waddling gait^29^. The experimental procedures were approved and performed in accordance with the guidelines of the National Taiwan University College of Medicine and the College of Public Health Institutional Animal Care and Use Committee (IACUC No. 20170457). PD mice were obtained by homozygous mating. Age- and sex-matched B6/129 hybrid mice were used as controls.

### MRI studies

All brain images of 9- and 18-month-old mice were acquired on a 7T preclinical MRI scanner (BioSpec 70/30 USR, Bruker Corporation, Billerica, MA) equipped with an ^1^H 2 × 2 mouse brain surface array coil and the ParaVision 5 software interface. After placing a mouse in the magnet isocenter, the B0 magnetic field was optimized by performing a field-map-based shimming (MAPSHIM software package of ParaVision 5.0). The T2-weighted imaging was performed using a rapid acquisition with relaxation enhancement (RARE) sequence^30^ with following parameters: repetition time (TR)/effective echo time (TE_eff_) = 2000 ms/26.7 ms, field-of-view (FOV) = 20 × 20 mm^2^, matrix = 256 × 256, 12 axial slices with 0.5 mm thickness, averages = 1, RARE factor = 8, flip angle = 90°, and in-plane resolution = 78 × 78 μm^2^. We performed specific absorption rate (SAR) calculation when testing the imaging parameters (e.g., TR, TE, matrix size, voxel size) in the first DTI experiment. We used a program provided by the BRUKER vendor to calculate duty cycle and SAR to ensure the safety of radiofrequency (RF) deposition, and then optimize DTI imaging parameters for the following routine DTI scan. Once the estimated duty cycle was under 40%, DTI imaging parameters we set were acceptable to scan rodent brains. The diffusion MRI data was acquired using a single-shot spin-echo echo-planar-imaging sequence with following parameters: TR/TE = 6750 ms/34.5 ms, FOV = 20 × 20 mm^2^, matrix = 75 × 75, 22 axial slices with 0.267 mm thickness, averages = 10, flip angle = 90°, in-plane resolution = 267 × 267 μm^2^, b-values for 42 diffusion gradient directions (1 b0/41 directions) = 0/1000 s/mm^2^, diffusion gradient duration (δ) = 2.112 ms; diffusion gradient separation (Δ) = 25.699 ms^31^. To quantify the thickness of the corpus callosum, we measured the thickness of the genu at the midline on one axial slice that best delineated the genu. The relative T2 signal intensity (SI) over the genu of the corpus callosum was measured by the ratio of the SI of the genu to the SI of the bilateral ventricles.

A total of 41 diffusion-weighted images (b = 1000 s/mm^2^) and one baseline image (b = 0 s/mm^2^) were processed by an in-house DTI toolkit in MATLAB, thereby producing 3-dimensional brain maps that comprised a series of DTI indices of AD, RD, MD and FA. For each DTI index, a total of 30 brain maps (from 7 control mice and 8 PD mice with two DTI acquisitions) were averaged to create a midpoint average brain template using the MATLAB function for a series longitudinal registration in SPM 12 (Wellcome Department of Cognitive Neurology, UK). Hence, we created four average brain templates for AD, RD, MD, and FA. This procedure also produced a deformation matrix that can normalize individual brains to share the same spatial image coordinates with the template. After spatial normalization, we referred to the anatomical location from the histological mouse brain atlas^32^ and placed one each ROI was selected manually for the medial (12 voxels), left lateral (15 voxels), and right (12 voxels) lateral corpus callosum on the average brain template. The ROIs were used to sample AD, RD, MD and FA values from all normalized brain maps using AveLI in SPM 12^33^. AveLI provided the mean value of a DTI index within each ROI, allowing us to calculate the difference in mean DTI values between PD and control mice over 9 months.

### Histological and immunohistochemical studies

Mice were perfused and fixed with 4% paraformaldehyde. For corpus callosum thickness counting, brain tissues were embedded in paraffin and cut into 5-μm serial coronal sections for H&E staining. The thickness of the corpus callosum was measured from + 0.38 mm to + 1.18 mm from the bregma. For immunostaining, brain tissues were embedded in paraffin and cut into 30-μm coronal sections and stained with an anti-oligo2 antibody (Merck Millipore, Darmstadt, Germany) labeled with Alexa Fluor 488 (Invitrogen).

### Ultrastructure analysis and image collection and analysis

The lateral corpus callosum of mice was rapidly dissected and immersed in 2% paraformaldehyde and 2.5% glutaraldehyde in 0.1 M cacodylate buffer at pH 7.4 overnight at 4 °C. Samples were processed as previously reported^34,35^. After washing three times with the same buffer, the samples were postfixed in 1% osmium tetroxide buffered with cacodylate for 1 h at room temperature, dehydrated through a graded series of ethanol and embedded in Spurr’s resin. Ultrathin sections were stained with uranyl acetate and lead citrate and examined using a Hitachi H-7100 electron microscope equipped with a Gatan 832 digital camera (Gatan, 86 Inc.). Nonoverlapping EM images of the coronal-sectioned corpus callosum were analyzed to determine the g-ratio as previously reported^36^. Images were processed with ImageJ software (https://imagej.nih.gov/ij/) with a plug-in (https://gratio.efil.de) that allowed randomly selected axons to be analyzed. The outer border (used to calculate myelin area) and inner border (used to calculate axon area) of myelin sheath were manually marked, and the g-ratio was calculated as the axonal diameter divided by the myelinated fiber diameter. A total of 300 axons from 3 different images from the control mice and 600 axons from 6 different images from the PD mice were analyzed.

### Statistical analysis

All data are presented as the mean ± standard deviation (SD). Statistical analyses were performed using SPSS Statistics Version 17.0 using a Student’s t-test (myelin area, axon area, g ratio) and the Mann–Whitney test. A value of *p* < 0.05 was considered significant.

## Supplementary information

Supplementary Information.

## Data Availability

Data produced and processed in this study are included in the published article. The datasets can be acquired from the corresponding author upon request.
