# Supplementary material for: Ultrastructural and diffusion tensor imaging studies reveal axon abnormalities in Pompe disease mice
Source: Sci Rep. 2020 Nov 19;10:20239. doi: 10.1038/s41598-020-77193-w (PMC7677380; doi:10.1038/s41598-020-77193-w)
Supplement: Supplementary file 1 — Supplementary Information. [file 41598_2020_77193_MOESM1_ESM.docx]

**Ultrastructural and diffusion tensor imaging studies reveal axon abnormalities in Pompe disease mice**

Ni-Chung Lee, Wei-Hao Peng, Li-Kai Tsai, Yen-Hsu Lu, Hao-Chun Wang, Yao-Chia Shih, Zeng-Xian Pung, Hsi-Yuan Hu, Wuh-Liang Hwu, Wen-Yih Isaac Tseng, Yin-Hsiu Chien

**Supplementary Information**

1. Figure S1


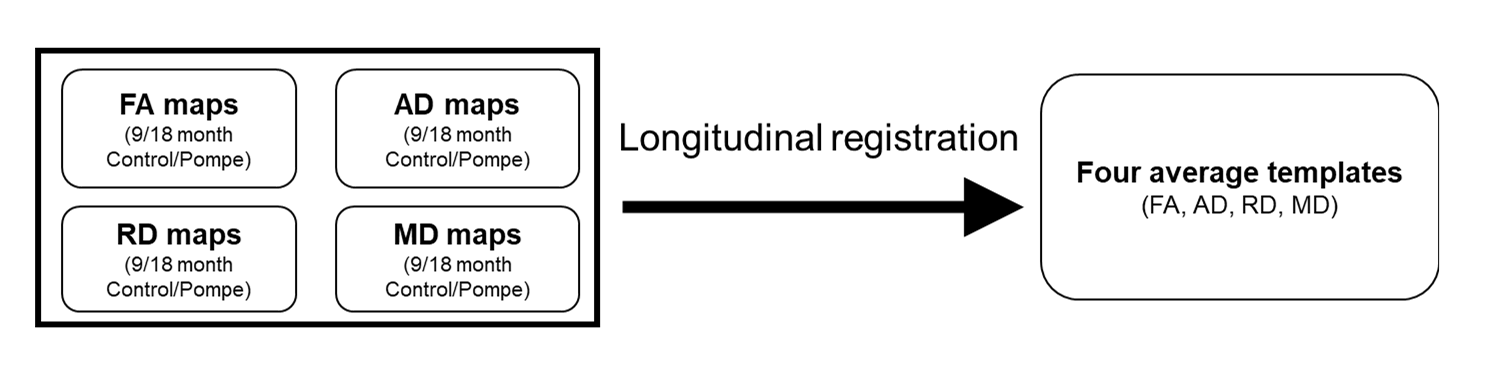


**Figure S1.** The flowchart of longitudinal registration to generate four average study-specific diffusion templates. All maps of control and Pompe mice across two examinations over 9 months were averaged to generate a study-specific template for each DTI metric via the longitudinal registration.

1.
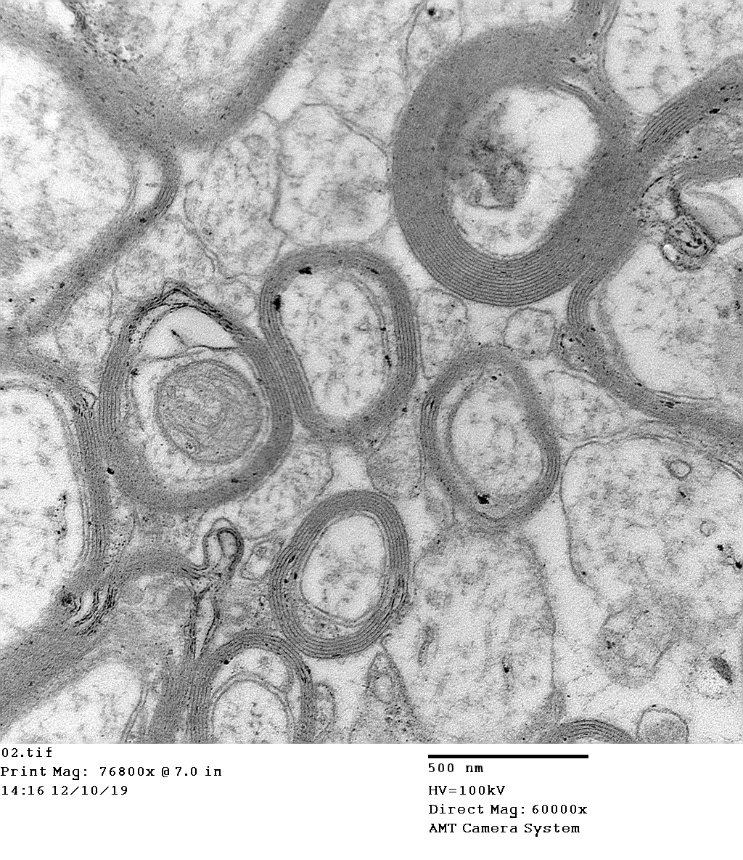
Figure S2

**Figure S2.** A high magnification image from a control mouse shows large and small nerve fibers but there is no splitting of the myelin sheath in the large fibers.
